# Corneal and conjunctival injury seen in urgent care centres in Israel

**DOI:** 10.1111/opo.12600

**Published:** 2019-01-10

**Authors:** Deena R Zimmerman, Einat Shneor, Michel Millodot, Ariela Gordon‐Shaag

**Affiliations:** ^1^ TEREM Emergency Medical Centers Jerusalem Israel; ^2^ Department of Optometry Hadassah Academic College Jerusalem Israel; ^3^ School of Optometry and Vision Sciences Cardiff University Cardiff UK

**Keywords:** ocular injury, urgent care centres, emergency department

## Abstract

**Purpose:**

Corneal and conjunctival injuries (CCI) comprise a large portion of the cases presenting to hospital‐based emergency departments (ED) with ocular involvement. Urgent Care Centres (UCC) offer community based emergency care at lower cost than hospital‐based emergency departments (ED) and with greater temporal convenience than primary care office settings. While CCI prevalence and treatment at hospital‐based EDs has been well studied, this is the first report, to our knowledge, on CCI demographics and aetiology presenting to UCCs.

**Methods:**

This retrospective study was approved by the institutional ethics committee. The setting is a UCC system in Israel, modelled on USA urgent care facilities, consisting of 17 branches at the time of the study. Electronic medical record data (between November 1, 2015 and October 31, 2016) of patients diagnosed with corneal disorder, foreign body or eye disorder were retrieved and reviewed for inclusion/exclusion criteria. Data collected included gender, age, chief complaint, diagnosis, treatment and discharge status (sent home or referred to ED). International Classification of Diseases, Ninth Revision (ICD‐9) codes were assigned to each record based on a review of all fields. UCC results were compared to all ED patients in Israel using data from a public report. Data were analysed by descriptive statistics and logistic regression analysis.

**Results:**

Of the 602 074 charts screened, 4797 patients presented with CCI (0.8%). The average age was 32.6 ± 18.2 years and 71.3% were male. Among these, 26.4% were referred to the ED compared to 6.8% from the entire UCC cohort. ICD‐9 code Foreign body (FB) of the eye was the most common cause of CCI (56.5%) followed by the following ICD‐9 codes: trauma (18.1%), chemical in the eye (11.1%) and corneal disorder due to a contact lens (5.1%). Logistic regression analyses showed the following risk factors for ED referral: age (22–64), male gender, ICD‐9 code FB, work‐related injury and the presence of a clinical abrasion in the eye.

**Conclusions:**

The aetiology of ocular injury at UCC is similar to previous studies of ED. Most CCI can be treated at UCC saving ED resources and underscores the importance of this mode of health care delivery in the overall health system.

## Introduction

Ocular injury is one of the leading causes of monocular blindness worldwide.^1^ In the USA 1.5% of all emergency department (ED) visits had an ophthalmic principal diagnosis,^2^ of which ~21% were due to corneal injury.^2^, ^3^ Several studies have found that between 23% and 44.4% of ocular injury cases in the ED were not an emergency and could have potentially been managed outside the ED.^2^, ^3^, ^4^, ^5^, ^6^, ^7^, ^8^, ^9^, ^10^, ^11^ Management of these cases outside the ED could make ED resources more available for emergency ophthalmic and medical issues.^3^

The last four decades has seen the rise of urgent care centres (UCC) to precisely treat such non‐emergency conditions.^12^ Urgent care refers to intermittent health care offered in the community for situations that need immediate attention but are not an emergency. Even though office‐based visits to ophthalmologists and optometrists could fit under this definition, office settings have delineated hours, while the need for treatment may arise outside of that framework. Furthermore, schedules may be filled in advance and lack the flexibility to allow additional appointments. In addition, community‐based office practices may lack certain diagnostic and treatment infrastructures. UCC offer walk‐in community‐based care at a lower cost than hospital‐based ED, while providing more ancillary services than the traditional practitioner's office. They also have greater temporal convenience than primary care office settings. UCC generally are not equipped to deal with trauma, provide resuscitation or admit patients to a hospital – all reasons for seeking ED care. This healthcare setting has grown worldwide,^12^ especially in countries such as Britain^13^ and the United States of America,^14^ where the concept of UCC was conceived and developed.

Treatment of ocular injury at UCC could reduce ED burden, but there is no research on this topic. This study describes the demographics of a cohort presenting to UCCs with corneal and conjunctival injuries (CCI) and compares it to the total cohort presenting to UCC as a control, as well as to patients presenting to Israeli ED during a similar period. Furthermore, it will describe the reasons for arriving to UCC, the aetiology of injury, the diagnosis and the risk factors for ED referral.

## Methods

### Study design

This anonymous retrospective study of electronic medical record data from the UCC database, was approved by the Hadassah Academic College Ethics Committee, and followed the principles of the Declaration of Helsinki.

### Setting and study population

The study setting is a nationwide UCC system,^12^ which had 17 branches at the time of the study. The four largest branches operated 24 hours a day, 365 days per year. Smaller branches were open evenings, weekends and holidays. All provided physical examinations by licensed physicians. Nursing duties were performed by either nurses or paramedics. Radiological and laboratory services were provided on site. In house training was provided to all nurses in visual acuity testing using a Snellen chart (without pinhole) and to all physicians in lid eversion and fluorescein staining followed by examination under cobalt blue light viewed with the naked eye. Physicians performed pupillary reaction in response to a swinging flashlight and ocular motility. The examinations were recorded in a uniform electronic medical record. TEREM quality assurance team aimed to ensure that the entire procedure was uniform at all branches.

Electronic medical records include (1) data from standardised lists: clinic location, visit date and time, demographics (gender and age), chief complaint, diagnosis (each electronic medical record may include several diagnoses), treatment(s), discharge prescriptions and discharge status (referred to ED or sent home) and (2) free text data: patient history, physical exam, assessment and treatment plan.

### Study protocol and measures

The study included all patients who presented to UCC in Israel between November 1^st^ 2015 and October 31^st^ 2016. The database was queried for all cases with the diagnosis of eye injury, foreign body in eye or ocular disorder. All records were reviewed and those who met the exclusion criteria were removed (*Table* 1).

**Table 1:** Exclusion criteria

Reason for exclusion	Explanation	No. of records excluded
Follow‐up	Patients who have presented to UCC for the same complaint in the past 48 hours	97
Missing data	Patients for whom there is not enough data to assign an ICD‐9 code	59
No data	Patients that do not have any data in clinical history, physical findings and summary	17
Peri‐ocular	Patients with problems of the ocular adnexa	9
Conjunctivitis	Conjunctivitis in the diagnosis and/or the history, exam and summary are consistent with conjunctivitis	28
Post‐op	Patients presenting for post‐operative care	4
Total		215

ICD, International Classification of Diseases.; UCC, urgent care centres.

The remaining cases were categorised according to ICD‐9 codes (International Classification of Diseases, Ninth Revision; *Table* 2). Other data extracted from the free text, when available, was the circumstances of the injury (particularly if work‐related) and if a corneal or conjunctival abrasion was observed with fluorescein staining. Demographics of the CCI cohort were collected and compared with that of the rest of the UCC.

**Table 2:** Urgent care centres (UCC) ocular problem categorization

ICD code name	ICD‐9 Code	Details
Abrasion in the conjunctiva, cornea, or eye	918.1,2	Corneal, conjunctival or non‐specified abrasion but no history of anything else such as FB or trauma
Corneal FB	930.0,1	FB in the cornea or conjunctival sac or patient reports a definite history of FB in the cornea (even if no FB found or abrasion observed)^a^
Chemical exposure	940.2,3 & 989.X	Cornea and conjunctival sac exposure to alkaline, acid or toxic chemicals
Other burn	940.4	Burn to cornea and/or conjunctival sac
Photokeratitis	370.24	Welder's keratitis or other form of photokeratitis (e.g. from a tanning bed)
Corneal disorder due to CL	371.82	Impacted contact lens or history that implicates CL in the pathology (for example slept in CL)
Trauma	959.0	Other and unspecified injury to head face and neck

CL, Contact Lens; FB, Foreign Body; ICD, International Classification of Diseases.

a The ‘feeling of FB’ or ‘felt FB’ was not classified as FB.

Data on all ED patients in Israel was collected from a public report of the State of Israel Ministry of Health Information Division. This report included the number of patients according to age group and gender who presented to all Israeli ED in the year 2015.^15^

### Data analysis

Descriptive statistics were used to describe the characteristics of all subjects according to gender, age group, ED referral and ICD‐9 code. To examine proportions between the nominal variables in the study (patients’ age) the Z‐proportion test was used. All tests were two‐tailed and values of *p* < 0.05 were considered statistically significant.

To examine the prediction of ED referral, a series of multivariate logistic regressions was performed to determine the odds ratio (OR) of the research variables, with the predictive variables in each regression model including patient age and gender for each injury type and the interaction between them. Goodness of fit was assessed with Nagelkerke R square. Analyses were performed in Microsoft Excel and SPSS version 24.

## Results

During the study period, 602 074 patients presented to UCC of which 4797 (0.8%) had CCI. The entire cohort presenting to UCC was used as a control and compared to the CCI cohort to determine the demographics of this sub‐population. To show differences between urgent and emergency care we also compared the CCI cohort to a national database of ED patients during a similar period.

*Table* 3 compares the percentage of patients in different age groups for the CCI, UCC and ED cohorts. When statistically significant, negative Z‐values show a lower percentage of CCI patients for a given age group in comparison to UCC/ED, while positive Z‐values show higher a percentage. The results show that compared to UCC, a higher percentage of CCI patients were in the 15–54 age group, while a lower percentage were in the 0–14 and 65 and older age groups. When patients with CCI are compared to those presenting to ED, a similar trend is observed. For the only ICD‐9 code that overlapped between CCI and the national ED database, foreign body (FB) in the eye, a similar age distribution was found with most of the patients between ages 22–64 (data not shown).

**Table 3:** Comparison of patients with corneal and conjunctival injuries (CCI), Urgent Care Centre (UCC) patients and Emergency Department (ED) patients by age group

Age group years	CCI		UCC		ED		Z‐test	
	*N*	%	*N*	%	*N* × 1000	%	Z‐value (CCI‐UCC)	Z‐value (CCI‐ED)
0	23	0.5	26 036	4.3	99	3.9	−12.96**	−12.16**
1–4	236	4.9	81 536	13.5	187	7.3	−17.40**	−6.39**
5–14	582	12.1	96 555	16.0	231	9.0	−7.34**	7.49**
15–17	228	4.8	25 525	4.2	77	3.0	2.06*	7.29**
18–21	340	7.1	35 867	6.0	190	7.4	3.19**	−0.79
22–34	1436	29.9	116 046	19.3	467	18.2	18.49**	20.97**
35–44	777	16.2	65 168	10.8	283	11.1	11.98**	11.23**
45–54	535	11.2	51 338	8.5	300	11.7	6.67**	−1.07
55–64	396	8.0	45 339	7.5	250	9.7	1.31	−3.97**
65–74	180	3.8	31 097	5.2	218	8.5	−4.35**	−11.67**
75–84	56	1.2	19 848	3.3	205	8.0	−8.12**	−17.35**
>85	8	0.2	7719	1.3	125	4.9	−6.72**	9.93**
All	4797	100	602 074	100	2563	100		

Z test, Z‐proportion test.

*p <* *0.05, **p <* *0.01.

Men comprised a larger percentage of CCI patients compared to UCC and ED cohorts (71.3% vs 50.2% and 49%). This is true for every age group aside from babies under a year of age (Table *S1*) and is significant for ages 15–75. Male CCI patients were significantly older than females (33.54 ± 17.29 vs 30.12 ± 20.03, *p* < 0.001; the median age for men was 31.88 and for women 27.02).

There was a significantly higher percentage of ED referrals from CCI than from UCC (26.4% vs 6.8%, *p* < 0.01, *Table* 4) and this was true for every age group aside from 75 to 84. The highest referral rates were for men between 22 and 74 years of age.

**Table 4:** Corneal and conjunctival injuries (CCI) and Urgent Care Centre (UCC) patients compared for Emergency Department (ED) referral by age group

Age group	CCI			UCC			Z‐test
	*N*	%	% Total	*N*	%	% Total	Z‐value (CCI‐UCC)
0	6	26.1	0.5	1823	7.0	4.5	3.58**
1–4	58	24.6	4.6	3359	4.1	8.2	15.75**
5–14	101	17.4	8.0	4528	4.7	11.1	14.32**
15–17	49	21.5	3.9	1284	5.0	3.1	11.23**
18–21	72	21.2	5.7	2191	6.1	5.4	11.46**
22–34	391	27.2	30.9	7357	6.3	18.0	31.80**
35–44	231	29.7	18.3	3832	5.9	9.4	27.39**
45–54	163	30.5	12.9	3589	7.0	8.8	20.86**
55–64	123	31.1	9.7	4022	8.9	9.9	15.29**
65–74	55	30.6	4.3	3704	11.9	9.1	7.70**
75–84	14	25.0	1.1	3438	17.3	8.4	0.60
>85	2	25.0	0.2	1675	21.7	4.1	2.20*
All	1265	26.4	100.0	40 801	6.8	100.0	53.15**

Z test, Z‐proportion test.

*p <* *0.05, **p <* *0.01.

In the CCI group, FB was the most common ICD‐9 diagnosis (*n* = 2709, 56.5%), followed by trauma (*n* = 870, 18.1%), chemicals in the eye (*n* = 528, 11.0%), welder's keratitis (*n* = 253, 5.3%), corneal disorder due to contact lens (*n* = 245, 5.1%), abrasions (*n* = 149, 3.1%) and burn (*n* = 43, 0.9%). When comparing by gender, more men than women presented with FB (79.2% men), trauma (62.1% men), welder's keratitis (99.2% men) and burns (72.1% men), while more women presented with corneal disorder due to a contact lens (63.3% women). Minimal differences between genders were seen with corneal/conjunctival abrasions (51.0% men) and chemicals in the eye (54.4% men).

Data regarding the type and activity at time of injury was extracted from the patient records, when available. The most common FB types were non‐metallic particles (10.4%), metal (10.3%) and building materials (6.9%). The most common activities resulting in FB, were angle grinding (8.8%) and leisure activities (2.6%). Chemicals in the eye were due to the use of household chemicals (32.4%), household cleaning solutions (30.1%) and work‐related chemicals (9.1%). Most cases of corneal disorder due to a contact lens were caused by impacted contact lenses (53.4%), over‐wear (19.6%) and pain following lens removal (13.9%). For ICD‐9 trauma code (Table *S2*), the most common causes of trauma were from body parts (e.g. hand, finger; 25.8%), household items (14.4%) and foliage (7.9%).

Stratifying referrals to ED by ICD‐9 code diagnosis, FB was referred most often (28.2%), followed by burn (27.9%), trauma (27.7%), abrasions (24.8%) corneal disorder due to contact lens (23.7%), chemicals in the eye (21.6%) and welders’ keratitis (15.4%).

Logistic Regression predicting ED referral as a function of ICD‐9 code, age group and gender showed that ‘goodness of fit’ was excellent for all conditions (*Table* 5). ICD‐9 code of FB was a significant risk factor for referral, especially for ages 22–64. The risk factors for referring men were similar to the entire cohort, however women of all ages presenting with FB were less likely than the entire cohort to be referred to ED. Trauma (ICD‐9 code 959.0; Table *S2*) was a significant risk for ED referral for the entire cohort, ages 5–21 and 65+. Similar risk factors for trauma referral were observed for men, but not for women. Certain ICD‐9 codes such as welders’ keratitis and contact lens related disorders were less likely to be referred to ED than other ICD‐9 codes.

**Table 5:** Logistic regression for predicting emergency department referral by ICD‐9 condition, age groups and gender in odds ratio (OR), values (1‐Yes, 0‐No for all categories)

	Age groups				Total (*N* = 4797)	Nagelkerke R²
	0–4 (*N* = 259)	5–21 (*N* = 1150)	22–64 (*N* = 3144)	65+ (*N* = 244)		
Men (*N* = 3421)						
ICD welder	2.94	0.14	0.40***	0.47	0.42***	0.011
ICD abrasion	1.48	2.02	0.61	0.60	0.83	0.000
ICD FB	0.68	0.74	1.59***	0.96	1.39***	0.007
ICD Chemical	1.17	0.67	0.69	0.68	0.67*	0.002
ICD CL	—	1.15	0.62	—	0.71	0.001
ICD burn	0.00	5.44*	0.38	—	0.73	0.000
ICD trauma	1.26	1.69**	1.03	2.88*	1.08	0.000
Women (*N* = 1376)						
ICD welder	—	—	—		3.80	0.001
ICD abrasion	1.24	1.23	1.01	3.96	1.26	0.001
ICD FB	0.80	0.69	0.71	0.68	0.70**	0.008
ICD chemical	2.17	0.65	0.76	0.39	0.83	0.001
ICD CL	—	1.68	1.21	0.97	1.27	0.002
ICD burn	6.89	—	1.26	—	2.74	0.003
ICD trauma	0.41	1.27	1.52*	2.07	1.27	0.003
Entire cohort (*N* = 4797)						
ICD welder	3.08	0.14	0.47	0.48	0.49***	0.006
ICD abrasion	1.34	1.47	0.67	1.66	0.92	0.000
ICD FB	0.74	0.73*	1.45***	0.85	1.24**	0.003
ICD chemical	1.63	0.65	0.63*	0.49	0.68***	0.002
ICD CL	0.33***	0.23***	0.41***	0.41***	0.86	0.000
ICD burn	1.23	5.66*	0.53	—	1.08	0.000
ICD trauma	0.78	1.55**	1.06	2.46*	1.09	0.000

Chemical, Chemical exposure to the eye; CL, Contact Lens; FB, Foreign Body; ICD, International Classification of Diseases. Goodness of fit was assessed with Nagelkerke R².

*p <* *0.05, **p <* *0.01, ***p <* *0.001.

Data extracted from records included whether the injury resulted in a clinical ocular abrasion. Ocular abrasion was observed in 51.1% (2451/4797) of the patients and not observed in 25.2% (1211/4797), while the rest did not have enough information for assessment (23.7%). To determine if the presence of an ocular abrasion was more likely to result in an ED referral, a logistic regression analysis of predicting the odds ratio (OR) was performed on the available data. Logistic regression analysis showed that abrasion patients were at a high risk (OR 2.06, *p* < 0.001, Z = 0.028) of ED referral. This was significant for both men (OR 1.97, *p* < 0.001, Z = 0.025) and women (OR 2.08, *p* < 0.001, Z = 0.028).

Data extracted from records included whether the injury was work‐related. Of all cases, 26.4% (1265/4797) were classifiable as work‐related and 38.3% (1838/4797) as not work‐related, with the remainder (35.3%) having insufficient data for classification. Logistic regression analysis showed that work‐related activities were a significant risk factor for the entire cohort for ED referral (OR 1.86, *p* < 0.001, Z = 0.027), and for men (OR 1.89, *p* < 0.001, Z = 0.027) but not for women (OR 1.58, *p* > 0.05, Z = 0.005).

Comparing the three groups for gender and age, showed that the percentage of men was 93.7%, 52.7% and 72.1%, for work‐related, not work‐related and unknown, respectively. The age of presentation at UCC was similar for work‐related and unknown injuries, while 95.2% of work‐related injuries and 93.3% of unknown were aged 15–64. Only 47.7% of non work‐related injuries belonged to this age cohort.

## Discussion

While previous research has described the epidemiology of CCI in hospital‐based emergency departments,^2^, ^3^, ^5^, ^6^, ^7^, ^9^, ^10^, ^11^ this is the first time this issue has been addressed at community based UCC. In this study, an injury to the cornea and/or conjunctiva was present in 0.8% of patients who presented to Urgent Care Centres (UCC) with about half of them presenting with a foreign body in the eye. The comparison of the results of this study to previous ED and hospital based studies (*Table S3*) shows similar trends: the majority of CCI occur to men,^2^, ^3^, ^4^, ^5^, ^6^, ^7^, ^8^, ^9^, ^10^, ^11^, ^16^, ^17^, ^18^, ^19^ tend to be work‐related,^6^, ^8^, ^17^, ^18^, ^19^, ^20^ involve foreign body in the eye^2^, ^3^, ^6^, ^9^, ^11^, ^16^ and angle grinding.^5^, ^9^, ^19^ Since UCC in Israel was modelled on that in the United States, and the aetiology of CCI are similar, it is reasonable to assume these finding can be extrapolated to other countries as well.

Urgent care centres were able to treat 74% of the CCI patients, including treatment of 72% of patients with FB, which is considered an emergency condition.^3^ These patients may have otherwise presented to the ED, increasing both ED burden and patient cost.^3^ The relative cost of ED to UCC depends on the health care system in question. In Israel, ED costs about three times as much as UCC,^12^ while in the USA, ED is approximately four times more expensive.^3^, ^21^, ^22^ This means that there are considerable savings when patients are treated at UCC. However, perhaps some of the savings is offset by the fact that the health care system may have to pay both UCC and ED for the 26% of the patients referred in this study. Using the relative cost of UCC vs ED cited above, there would still be a savings of 60% in Israel and 52% in the USA. These findings emphasise the importance of UCC in healthcare systems. The percentage of CCI patients sent home, however, is lower than that for total UCC (93%). Fewer ED referrals may have been needed if UCC had more ophthalmic equipment and an ophthalmologist or optometrist on call. However, considering the relatively small number of CCI visits, this may not be cost effective.

More men with CCI were referred to ED than women (77.3% vs 22.7%) with the largest difference found between the ages of 18 and 64 (*p* < 0.01). In contrast, the percentage of ED referrals from the total UCC cohort was almost equal between the genders. This may be due to the aetiology of injury: men are more likely to be referred for work‐related FB injury and women are more likely to be referred with trauma that is not work‐related. The present results are similar to those reported in many other ED based study of ocular injuries (*Table S3*).

Twenty six percent of the patients had a clear work‐related injury. For 35% of the patients, the records had insufficient information to determine whether it was work‐related or not. The demographics of this unknown group suggest that some of them may be work‐related. This cohort of patients resembles the work‐related cohort in term of age. In terms of gender, the percentage of men (72.1%) is somewhere between the work‐related (93.7%) and non‐work‐related (52.7%) groups. These finding indicate that the actual number of work‐related injuries may be larger but requires further investigation.

Our findings may indicate that proper use of protective eyewear during work‐related activities could reduce the number of CCI, especially FB. Similarly, a study of stone‐quarry workers demonstrated the effectiveness of protective eyewear in reducing injury.^15^ To that end, there are national directives in Israel regarding use of safety goggles in welding, angle grinding, carpentry and other types of work that would endanger the eyes and face.^23^ The directives require the employer to provide protective eyewear and the worker to use it. Nevertheless, many of the patients in this study were not likely to have used protective eyewear during work‐related activities. However, the patients were not asked about protective eyewear, so this requires further investigation. The results of this study indicate that both workers and employers may benefit from education about the benefits of occupational safety.

Several common ocular conditions were not included in this study. Injuries such as orbital fractures, which are a common ED occurrence, especially in the elderly^2^, ^3^ were not reported in the present study. Most likely, the search terms used in this study would not pick up orbital injuries. In addition, patients with more extensive injuries involving the eye probably go directly to a hospital ED. Subconjunctival haemorrhages, which are common in the ED, were also not reported in this study. The search term ‘subconjunctival hematoma’ in the diagnosis field was not used so that we could compare our results to previous publications describing ocular injury in emergency departments such as Vasiri *et al*.^2^ which do not categorise subconjunctival hematomas as injury. The term subconjunctival hematoma did appear several times together with eye injury and/or eye disorder in the diagnosis term. In those cases, the file was only included if it met the inclusion criteria.

### Limitations

The main limitation of this study was its retrospective nature resulting in missing data in some of the records.

## Conclusion

Urgent Care Centres are an important component of the spectrum of patient care that can significantly save on healthcare costs and reduce overloading hospital emergency departments. Men with foreign body injuries may benefit by presenting directly to the ED, if possible given the guidelines of the healthcare reimbursement system. This can impact emergency and urgent care worldwide.

## Disclosures

Deena R. Zimmerman is an employee of TEREM in the capacity of the Director of Research and a Medical Director at TEREM but has no conflict of Interest. Einat Shneor, Michel Millodot and Ariela Gordon‐Shaag report no conflicts of interest and have no proprietary interest in any of the materials mentioned in this article.

## Supporting information


**Table S1.** Comparison of corneal and conjunctival injuries (CCI), urgent care centres (UCC) and emergency department (ED) by age group and gender

**Table S2**. Causes of trauma (ICD‐9 code 959.0) by gender

**Table S3**. Ocular injury reported in various studies
