# Supplementary material for: Corneal and conjunctival injury seen in urgent care centres in Israel
Source: Ophthalmic Physiol Opt. 2019 Jan 10;39(1):46–52. doi: 10.1111/opo.12600 (PMC6850452; doi:10.1111/opo.12600)
Supplement: Supplementary file 1 — Table S1. Comparison of corneal and conjunctival injuries (CCI), urgent care centres (UCC) and emergency department (ED) by age group and gender [file OPO-39-46-s001.docx]

**Supplementary Table 1.** Comparison of CCI, UCC and ED by Age Group and Gender

Abbreviations: Corneal and Conjunctival Injuries (CCI), Urgent Care Centres (UCC), Emergency Departments (ED), Z-proportion test (Z test).

| **Z-test** | | | **ED** | | **UCC** | | **CCI** | | **Age group** |
| --- | --- | --- | --- | --- | --- | --- | --- | --- | --- |
|  | Z-value (CCI-ED) | Z-value (CCI-UCC) | %Male | N x 1000 | %Male | N | %Male | N |  |
|  | -0.47 | -1.47 | 44 | 99 | 54.4 | 26,036 | 39.1 | 23 | **0** |
|  | 3.03** | -0.33 | 44 | 187 | 54.9 | 81,536 | 53.8 | 236 | **1-4** |
|  | 11.31** | 1.8 | 39 | 231 | 58.2 | 96,555 | 61.9 | 582 | **5-14** |
|  | 6.82** | 4.01** | 45 | 77 | 54.2 | 25,525 | 67.5 | 228 | **15-17** |
|  | 7.85** | 7.32** | 45 | 190 | 46.3 | 35,867 | 66.2 | 340 | **18-21** |
|  | 15.33** | 21.46** | 54 | 467 | 45.8 | 116,046 | 74.2 | 1,436 | **22-34** |
|  | 16.26** | 16.4** | 50 | 283 | 49.6 | 65,168 | 79.2 | 777 | **35-44** |
|  | 14.39** | 13.77** | 47 | 300 | 48.2 | 51,338 | 78.1 | 535 | **45-54** |
|  | 11.47** | 11.76** | 47 | 250 | 46.2 | 45,339 | 75.8 | 396 | **55-64** |
|  | 4.13** | 5.27** | 49 | 218 | 44.8 | 31,097 | 64.4 | 180 | **65-74** |
|  | -0.6 | 1.21 | 54 | 205 | 42 | 19,848 | 50 | 56 | **75-84** |
|  | -0.52 | 0.42 | 59 | 125 | 42.6 | 7,719 | 50 | 8 | **>85** |
|  | 30.87** | 29.11** | **49** | **2,563** | **50.2** | **602,074** | **71.3** | **4,797** | **All** |

* p<.05, **p<.01
