# Supplementary material for: Corneal and conjunctival injury seen in urgent care centres in Israel
Source: Ophthalmic Physiol Opt. 2019 Jan 10;39(1):46–52. doi: 10.1111/opo.12600 (PMC6850452; doi:10.1111/opo.12600)
Supplement: Supplementary file 2 — Table S2. Causes of trauma (ICD‐9 code 959.0) by gender [file OPO-39-46-s002.docx]

**Supplementary table 2.** Supplementary table 2 – Causes of Trauma (ICD-9 code 959.0) by Gender

| **CCI** | | | **Type of Trauma** |
| --- | --- | --- | --- |
| % male | % | N |  |
| 52.2% | 25.8% | 224 | Body part (e.g. finger, hand, foot, etc…) |
| 57.6% | 14.4% | 125 | Household items |
| 66.7% | 11.4% | 99 | No data |
| 66.7% | 7.9% | 69 | Foliage |
| 62.1% | 7.6% | 66 | Ball/play |
| 71.9% | 7.4% | 64 | Attack |
| 72.6% | 7.1% | 62 | Materials: sticks, stone, metal, plastic |
| 72.2% | 6.2% | 54 | Other |
| 45.9% | 4.3% | 37 | Office supplies |
| 90.6% | 3.7% | 32 | Work-related instruments |
| 68.2% | 2.3% | 22 | Fall |
| 43.8% | 1.8% | 16 | Animal |
| **62.1%** | **100%** | **870** | **Total** |
