# Supplementary material for: Corneal and conjunctival injury seen in urgent care centres in Israel
Source: Ophthalmic Physiol Opt. 2019 Jan 10;39(1):46–52. doi: 10.1111/opo.12600 (PMC6850452; doi:10.1111/opo.12600)
Supplement: Supplementary file 3 — Table S3. Ocular injury reported in various studies [file OPO-39-46-s003.docx]

**Supplementary Table 3**. Ocular Injury Reported in Various Studies

Abbreviations: Ophthalmology (OMD), Ophthalmology, Foreign Body (FB), Emergency Department (ED).

| Study (Country) | Nature of the study | Sample size | Location | Male (%) | Aetiology/ risk factor |
| --- | --- | --- | --- | --- | --- |
| Baker 1996(16)  (USA) | Work-related injury | 1876 | Hospital admissions | 93.6 – work -related  74 – not work- related | 14% work-related |
| Nash 1998(13)  (USA) | Injury of eye and Ocular Adnexa | 2.3X10^6^ | ED | 63.1 | FB second most common diagnosis;  17.9 % work- related |
| Oum et al 2004(7) (Korea) | Ocular Trauma | 1809 | ED | 65.4 | Metal most common FB |
| Cillino 2008(12)  (Italy) | Ocular Trauma | 298 eyes | Hospital Dept. of OMD* | 84.6 | In Men: outdoor activities, work-related and sports  Women: home and outdoor activities |
| Saeed 2010(14)  (Ireland) | Ocular Injury | 517 | Patients hospitalized at OMD dept. | >75 each year | 49% work-related |
| Jafari 2010(5) (Iran) | Ocular trauma | 1950 | ED | 87.6 |  |
| Soong 2011(15)  (Malaysia) | Ocular trauma | 546 | Dept. Of OMD | 88.1 | 30.8% High powered tools  43.6% work-related |
| Northey 2014 (6) (Australia) | Ocular Trauma | 411 | ED | 77.9 | Work-related: angle grinding, FB |
| Desai 2015(3) (Scotland) | Ocular Trauma | 1728 | ED | 85 | Assault, machine tools |
| Cai 2015(11)  (China) | Work-related trauma | 1055 | Out patients and in patients OMD Hospital dept | 80.3 | FB most prevalent |
| Vaziri 2016(9)  (USA) | All eye related Visits | 1.996X10^6^  1.5% of all visits | ED | 53.2 | 21% Corneal injury with or without FB |
| Channa 2016(2)  (USA) | All eye related visits  Emergent vs. non-emergent | 12 X10^6^ | ED | Emergency visits risk factor | 21% Corneal injury with or without FB |
| Haring 2016 (USA) (4) | Ocular trauma | 5 X10^6^ | ED | 64.8 | 23% FB for men  Activities:  Men: outdoor, work and sports  Women: home and outdoor activities |
| Stagg 2017(8) (USA) | Emergency vs. non-emergency | 376680 | ED | Male sex risk factor |  |
| Current study  (Israel) | Injury to cornea and conjunctiva | 602,073 | UCC | 71.3 | ~50% FB |
